# Reticulocyte Hemoglobin-Equivalent Potentially Detects, Diagnoses and Discriminates between Stages of Iron Deficiency with High Sensitivity and Specificity

**DOI:** 10.3390/jcm11195675

**Published:** 2022-09-26

**Authors:** Majed N. Almashjary, Ahmed S. Barefah, Salem Bahashwan, Ibraheem Ashankyty, Refaat ElFayoumi, Majed Alzahrani, Duaa M. Assaqaf, Raghad S. Aljabri, Amera Y. Aljohani, Rema Muslim, Sara A. Baawad, Waleed M. Bawazir, Saif A. Alharthy

**Affiliations:** 1 Department of Medical Laboratory Sciences, Faculty of Applied Medical Sciences, King Abdulaziz University, Jeddah 22254, Saudi Arabia; 2 Hematology Research Unit, King Fahd Medical Research Center, King Abdulaziz University, Jeddah 22254, Saudi Arabia; 3 Department of Hematology, Faculty of Medicine, King Abdulaziz University, Jeddah 22254, Saudi Arabia; 4 Department of Medical Laboratory Technology, Faculty of Applied Medical Sciences in Rabigh, King Abdulaziz University, Jeddah 22254, Saudi Arabia; 5 Zoology Department, Faculty of Science, Mansoura University, Mansoura 35516, Egypt; 6 Hematology Laboratory, King Abdulaziz University Hospital, King Abdulaziz University, Jeddah 22254, Saudi Arabia; 7 Department of Internal Medicine, Faculty of Medicine, King Abdulaziz University, Jeddah 22254, Saudi Arabia; 8 Toxicology and Forensic Sciences Unit, King Fahd Medical Research Center, King Abdulaziz University, Jeddah 22254, Saudi Arabia

**Keywords:** iron deficiency anemia, iron deficiency, reticulocyte hemoglobin equivalent, iron, diagnosis

## Abstract

Background: Iron deficiency anemia (IDA) is a global health problem affecting the quality of life of more than 2 billion individuals. The current practice guidelines diagnose and monitor IDA via conventional hematological and iron biomarkers, which take several months before they are corrected under an iron-treatment plan. Reticulocyte hemoglobin equivalent (Ret-He) is used as a marker in most new hematology analyzers to assess iron incorporation into erythrocyte hemoglobin directly. This study aims to examine the efficacy of Ret-He as a marker for iron deficiency (ID) and IDA and investigate whether Ret-He is sensitive to iron therapy. Methods: Two blood samples were drawn from 182 participants for CBC and iron profile measurements. Follow-up samples were drawn from participants with a confirmed diagnosis of ID and/or IDA. Results: Ret-He levels were lower in the ID and IDA groups compared to the control (*p* < 0.0001), and lower in the IDA group compared to the ID group (*p* < 0.0001). Ret-He was correlated with ferritin at ID level (<30.0 mg/mL; r = 0.39) and severe IDA (<13.0 ng/mL; *p*-value < 0.01, r = 0.57). Cut-off values of <28.25 pg for ID and <21.55 pg for IDA showed a higher specificity and sensitivity (ID; AUC: 0.99, sensitivity: 92.73%, specificity: 97.87%) and (IDA; AUC: 0.94, sensitivity: 90.63%, specificity: 92.31%). Finally, Ret-He successfully reflected the iron therapy (*p* < 0.001) when compared to hemoglobin (Hb) (*p* = 0.1). Conclusions: Ret-He is a potential marker for detecting and diagnosing different stages of ID with high validity and is very sensitive in reflecting the iron incorporation in a short time.

## 1. Introduction

Anemia is a pervasive global health problem with an estimation of 2 billion people affected with it [1], and it was accountable for more than 68 million years lived with disability (YLD) worldwide [2]. Iron deficiency anemia (IDA) is considered the leading cause of anemia in males and females [3]. IDA prevalence is highly increased in women of child-bearing age and preschool children at a range between 30 and 50% [1,4]. The global distribution of anemia is unequally dispersed, especially in undeveloped regions, where the anemic cases are five times higher [5]. In Saudi Arabia, iron deficiency anemia is common, especially among children and women of child-bearing age [6]. Several studies conducted in the Saudi population were directed mostly to assess the prevalence of IDA in children and women of reproductive age, with a prevalence of 30 to 60% [4,7,8,9].

Iron deficiency anemia can be defined as a condition where erythropoiesis is restricted, affecting the production of mature red blood cells (RBCs) due to diminished iron levels. As a result of erythropoiesis restriction, the red cell mass and hemoglobin (Hb) carrying capacity decrease [10]. Several factors primarily affect the amount of iron, such as blood loss, malnutrition, fetal-maternal bridge, and some common parasitic infections in the underdeveloped countries for preschool children and women of reproductive age [5,6,7,8,9,10].

Iron deficiency anemia can be diagnosed through several hematological and biochemical biomarkers including Hb, mean cell volume (MCV), mean cell hemoglobin (MCH), red cell distribution width (RDW), serum ferritin, serum iron, transferrin saturation (TS), total iron binding capacity (TIBC), and soluble transferrin receptor (sTfR) [11]. Nevertheless, the gold-standard approach is bone marrow (BM) iron deposition that qualitatively measures the iron available for erythropoiesis. The caveats with this approach are its invasiveness and need of expertise in acquiring, handling, and staining bone marrow aspirates [12]. The non-invasive method currently used by most laboratories is the serum ferritin concentration to indicate the body’s iron store [13], providing reliable results, similar to those of the invasive gold-standard method [14].

The duration of reticulocyte maturation from being synthesized in the bone marrow until they become mature RBCs is 3 to 4 days, half of it spent in the peripheral blood to become mature RBCs. Therefore, using the blood sample is helpful to analyze and diagnose the iron levels via reticulocytes [15]. The assessment of the hemoglobin content in reticulocytes reflects the iron levels [16,17]. New hematology analyzers, such as Sysmex XE and XN models, Siemens ADVIA, and Alinity h series, can measure the hemoglobin content in reticulocytes through the principle of fluorescence flow cytometry.

Reticulocyte-hemoglobin equivalent (Ret-He) can detect iron therapy’s response because the reticulocyte has a short life [18]. Using Ret-He as a biomarker is advantageous over other hematological biomarkers, for which the iron treatment would take several weeks or months to show response [19,20]. Accordingly, this study aims to examine the efficacy of Ret-He as a marker for iron deficient (ID) and IDA and investigate whether Ret-He is sensitive to iron therapy in ID and IDA patients.

## 2. Materials and Methods

### 2.1. Study Participants

This study was conducted at King Fahd Medical Research Center and was approved by Biomedical Ethics at the Faculty of Medicine and King Abdulaziz University Hospital (No. 185-21). The recruitment of study participants was based on a circulating questionnaire among the general population. Written consent was acquired from all the participants. Participants who were pregnant, on iron supplementation at the time of enrollment, had a blood transfusion in the last three months, or suffered from hemoglobinopathies or malignancy were excluded from the study.

### 2.2. Blood Collection and Measurements

Two blood samples were collected using two different tubes. One sample was collected from participants in an ethylenediamine tetraacetic acid dipotassium salt (EDTA-2K) tube for complete blood count (CBC) and reticulocyte profile using a modern automated hematology analyzer, Sysmex XN-9000 (Sysmex Corporation, Kobe, Japan). Another blood sample was collected into a plain gel-separator tube to measure the iron biomarkers (serum ferritin, TIBC, and serum iron). These samples were centrifuged at 3500× *g* rpm for 5 min to separate the serum and then were run on the Alinity system (Abbott Laboratories, Chicago, IL, USA) that utilizes chemiluminescent microparticle immunoassay (CMIA). The transferrin saturation (TS) was calculated using the following formula: (Serum iron/TIBC) × 100.

Follow-up blood samples were collected from participants who met the criteria for ID or IDA (*n* = 28) on the 7th day after the daily oral administration of 190 mg of ferrous sulfate.

### 2.3. Definition of Anemia & Iron Deficiency Anemia

Following WHO guidelines, the diagnosis of anemia was applied when the Hb level was lower than 13 g/dL for men and less than 12 g/dL for women [21]. A serum ferritin level of <30 ng/mL with normal hemoglobin was considered an insufficient iron store or iron deficiency (ID). When ID was coupled with a Hb concentration of <13 g/dL for men and <12 g/dL for women, it was categorized as iron deficiency anemia (IDA). Participants with normal-to-high serum ferritin and low hemoglobin were considered to have anemia without iron deficiency (non-IDA).

### 2.4. Statistical Analysis

All statistical analysis and graphing were conducted using the GraphPad prism 9 software (GraphPad Software, San Diego, CA, USA). The data obtained from all the study groups were not normally distributed and therefore compared using the Kruskal–Wallis test. Dunn’s post-hoc test was used for the multiple comparisons, and the Wilcoxon matched pairs signed rank test was used to examine the effect of the treatment on the investigated markers. A spearman correlation was used to assess the association of Ret-He with other iron markers, while the sensitivity and specificity of Ret-He as a marker were determined by using receiver-operating characteristic (ROC) plots. A *p*-value of <0.05 was considered statistically significant.

## 3. Results

### 3.1. Participants’ Demographic and Clinical Information

A total of 182 participants were enrolled in the study. The control group was composed of 87 healthy individuals from both genders who had ferritin > 30 ng/mL and Hb > 13.0 and >12.0 for males and females, respectively. Nearly all participants in the control group were in the age between 18 and 29 years (97.7%). The remaining 95 participants were divided between the three study groups, ID, IDA, and non-IDA, according to their ferritin and hemoglobin levels as described earlier. A total of 9 males were in two of the study groups, ID (14.5%) and non-IDA (12.5%), and none in the IDA group. The majority of female participants were in the ID and IDA groups with a percentage of 85.5% and 100%, respectively, and the rest (*n* = 7) were in the non-IDA group (Table 1).

Next, selected hematological parameters related to RBC size and hemoglobin content (Hb, MCV, MCH, MCHC, RDW and Ret-He) and iron biomarkers (ferritin, serum iron, TIBC, and TS) were compared between the four groups using the Kruskal–Wallis test. A strong significant difference was observed among all the four groups, as shown in Table 2.

**Figure 1:**
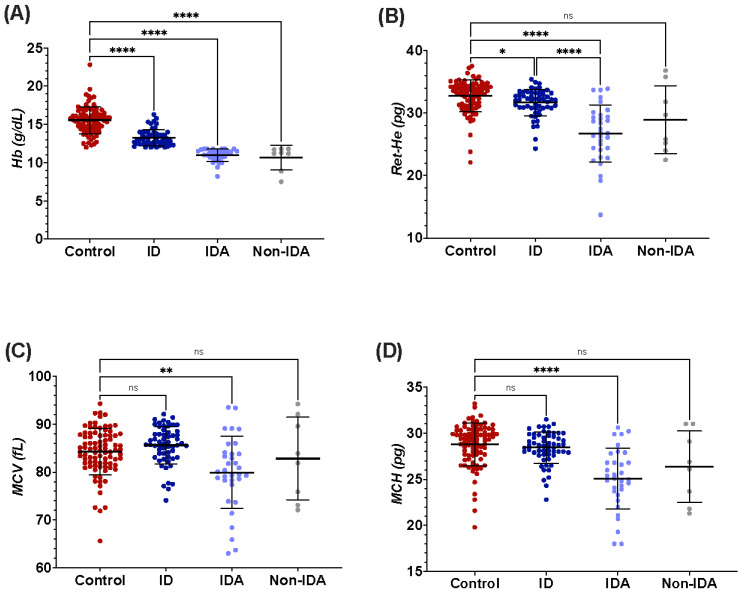

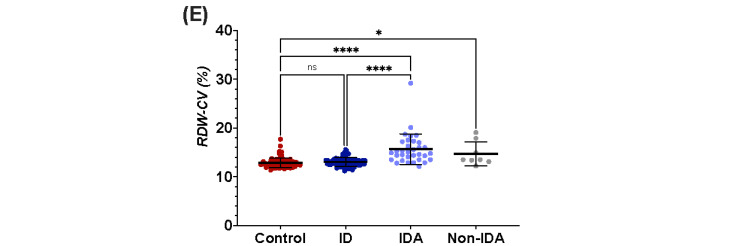
Plots for hematological parameters showing multiple comparisons between the four groups based on their iron levels using Dunn’s post-hoc. Horizontal and error lines represent mean and SD, respectively. * A *p*-value of <0.05 was considered statistically significant, ** for a *p*-value of <0.01, **** for a *p*-value of <0.0001, and (ns) for non-significant. (**A**) Multiple comparison plots for hemoglobin (Hb), (**B**) reticulocyte hemoglobin equivalent (Ret-He), (**C**) mean cell volume (MCV), (**D**) mean cell hemoglobin (MCH), and (**E**) red cell distribution width (RDW).

A post-hoc Dunn’s test was used to compare each of the experiment groups to the control. All the three experiment groups had significantly lower hemoglobin levels when compared to the control group (*p* < 0.0001 *p* < 0.0001, and *p* < 0.0001), including the ID group who had a hemoglobin level above the WHO cut-off values (*p* < 0.0001) (Figure 1A). The MCV and MCH were significantly decreased in the IDA group only when compared to the control (*p* < 0.01, and *p* < 0.001, respectively). The ID and non-IDA had unchanged levels of MCV and MCH, compared to the control group (MCV: *p* = 0.26, and 0.9, and MCH: *p* = 0.47, and 0.18); Figure 1C,D. The reticulocyte hemoglobin equivalent was significantly lower in the three experiment groups compared to the control (*p* < 0.05 for ID and non-IDA), with a significant reduction observed in the IDA group (*p* < 0.0001); Figure 1B.

**Figure 2:**
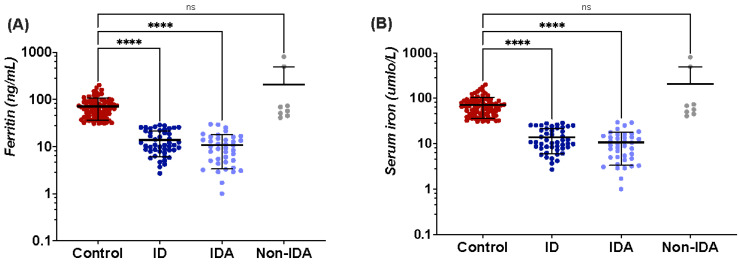

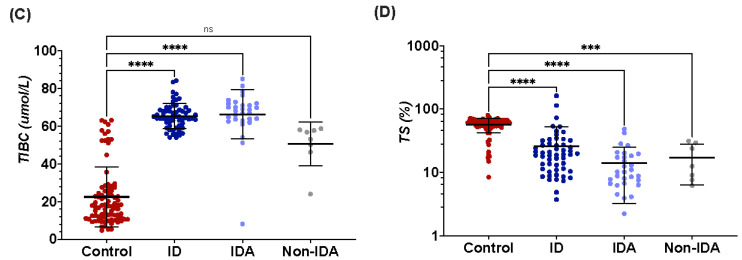
Plots for iron parameters showing multiple comparison between the four groups based on their iron levels using Kruskal–Wallis post-hoc test. Horizontal and error lines represent mean and SD, respectively. *** A *p*-value of <0.001 was considered statistically significant, **** for a *p*-value of <0.0001, and (ns) for non-significant. (**A**) Multiple comparison plots for ferritin concentration, (**B**) serum iron, (**C**) total iron binding capacity (TIBC), and (**D**) transferrin saturation (TS).

As expected in cases of iron insufficiency, the iron biomarkers (serum ferritin, serum iron, TIBC) were significantly decreased in the ID and IDA groups (*p* < 0.0001), while TS was increased when aligned versus the control group (*p* < 0.0001) (see Figure 2A–D). When the cause of anemia was not iron related, the iron biomarkers were not significantly changed from the control group, with the exception of transferrin saturation, where the level was significantly decreased compared to the control group (*p* < 0.001); Figure 2A–D.

### 3.2. Relationship between Ret-He and Iron Biomarkers

**Figure 3:**
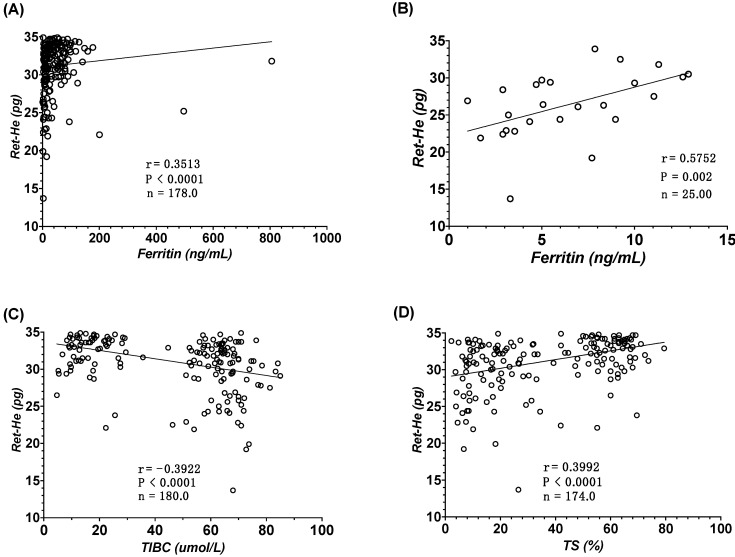
Correlation between reticulocyte hemoglobin equivalent (RET-He) and parameters of iron metabolism. (**A**) Correlation between RET-He and ferritin, (**B**) correlation between RET-He and ferritin < 13.0 ng/mL. (**C**) Correlation between RET-He and total iron binding capacity (TIBC), and (**D**) correlation between RET-He and transferrin saturation (TS).

To examine the relationship between Ret-He and markers for iron metabolism, a spearman correlation was performed on all data points. A modest positive correlation between Ret-He with ferritin (r = 0.35, *p* < 0.0001; Figure 3A), and TS (r = 0.39, *p* < 0.0001, Figure 3D) was observed. Ret-He was negatively correlated with TIBC (r = −0.39, *p* < 0.0001, Figure 3C). To further investigate the relationship between Ret-He and ferritin in severe cases of IDA, the ferritin threshold was set at <13 ng/mL. A strong significant and positive correlation was observed, where *p* = 0.002 and r = 0.57 as shown in Figure 3B.

### 3.3. Ret-He as a Diagnostic Marker for ID and IDA

**Figure 4:**
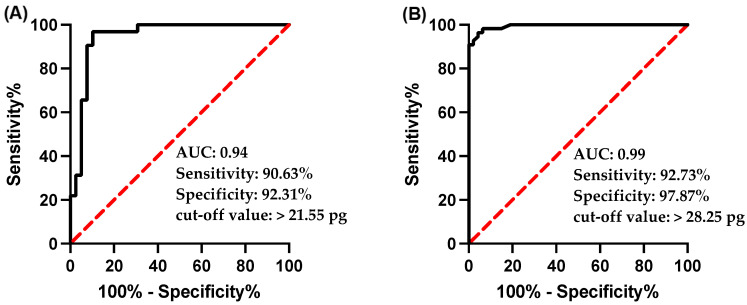
Receiver-operating characteristic (ROC) analysis of reticulocyte hemoglobin equivalent (RET-He) in the diagnosis of (**A**) IDA, and (**B**) ID.

The utilization of Ret-He as a diagnostic marker for ID and IDA when ferritin level < 30 ng/mL was assessed by ROC analysis and then plotted in Figure 4A,B for sensitivity versus specificity. The area under the curve (AUC) was 0.94 in the IDA group and the cut-off value of >21.55 pg had a sensitivity and specificity of 90.63% and 92.31%, respectively. For the usefulness of Ret-He as a marker for ID, the AUC was 0.99 and a cut off-value of >28.25 pg showed 92.73% sensitivity and 97.87% specificity.

### 3.4. Ret-He as a Marker for Detecting Early Iron Deficiency

The usefulness of Ret-He to detect early stages of ID was further analyzed against red cell distribution width (RDW), the marker that usually detects the latent ID. In this study, Ret-He outperformed RDW in detecting early stages of ID from the control (RDW: *p* = 0.5; Ret-He: *p* = 0.03) as shown in Figure 1B,E.

### 3.5. Ret-He as Marker for Response to Iron Therapy

**Figure 5:**
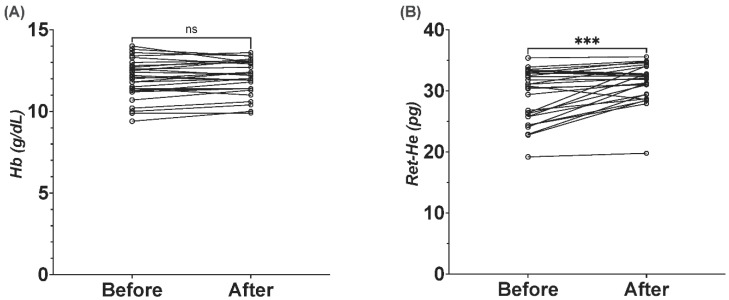
Time-course variation in hemoglobin, and reticulocyte hemoglobin equivalent in participants receiving iron treatment (190 mg ferrous sulphate) for one week. The graph on the left shows that hemoglobin did not change after one week of iron treatment (**A**), whereas Ret-He (**B**) had significantly increased following the treatment. Wilcoxon matched-pairs signed rank test was used to calculate the *p*-value. *** A *p*-value of <0.05 was considered statistically significant, and (ns) for non-significant.

To evaluate the efficacy of Ret-He as a marker for iron therapy response, participants who had ferritin concentration > 30 ng/mL were prescribed a daily tablet of 190 mg ferrous sulphate. Follow-up samples were drawn after one week from the participants. In comparison with the baseline measurement, Ret-He was significantly increased by 7.5% [29.2 *±* 4.3 vs. 31.4 *±* 3.2, *p* = 0.0002] (Figure 5B), indicating a successful iron incorporation into the erythron. Hemoglobin concertation did not respond to iron therapy, as the baseline mean of Hb concentration was 11.9 *±* 1.2 g/dL and only increased by 1.3% to reach a mean of 12.06 *±* 1.05 g/dL after a week on the treatment (Figure 5A).

## 4. Discussion

Iron deficiency is a global health problem that poses a major setback for the developing countries [1,3,4,5]. While the deprived iron storage and anemia could be adjusted with simple iron supplementation [5], it may take up from several weeks to months for the diagnostic markers to be corrected. The massive prevalence of ID and IDA globally demands for better markers that reflect the changes of iron therapy in a shorter period.

Reticulocyte has a short lifespan before it becomes mature [22]. Utilizing this small window to examine the effect of iron therapy would facilitate the decision to evaluate the treatment option rather than waiting for the treatment course to finish, which may take up to three months [23]. Therefore, this study aimed to evaluate the Re-He as a potential ID and IDA marker and its efficacy to reflect the response to iron supplementation after a week in the therapy.

The study examined 182 participants mostly in the age group between 18 and 29 years of age. A cut off < 30 ng/mL for serum ferritin was used, as it has a very high sensitivity (92%) and specificity (98%) to detect and diagnose overt ID [11,24,25]. In concordance with previous global and regional reports regarding the matter, majority of female participants in our cohort were either in ID (47.9%) or IDA (32.6%) groups [1,5,8,9,11,25,26,27,28,29,30,31,32].

Re-He can be a useful marker to distinguish between IDA and ID. In this study, the Re-He was found to be decreased in ID and IDA groups when compared to the control group, as the iron body store gets depleted, erythropoiesis becomes restricted and the amount of hemoglobin synthesized decreased in the red cell [33,34]. Importantly, Ret-He was significantly reduced in individuals with IDA in comparison to those with ID (*p* < 0.0001), suggesting that Ret-He is useful in distinguishing between iron store depletion and the presence of anemia. Several studies also showed that hemoglobin content in the reticulocytes has the capability to differentiate between these two conditions in adults [34,35] and pediatric patients [18]. The mean Ret-He in the ID and IDA groups was higher than what has been reported previously [34,35]. This can be attributed to two different factors: the different cut-off of ferritin level to define depleted iron stores, as Toki et al. used a <12.0 ng/mL definition, whereas Uçar et al. utilized a cut-off of <26.96 pmol/L. The other factor is the age difference in the studied populations as most of the participants in this study were college students between the ages of 18–29 years old, while the mean age in the other two studies was 40–50 years old [34,35], or in the pediatric population as reported by Brugnara et al. [18].

Since Ret-He strongly reflects the iron assessment into the erythron, we hypothesize that a shortage in the iron supply to erythropoiesis will be reflected before the other markers are affected. The ability of Ret-He to discriminate between the stages of ID and IDA was further evaluated against RDW, the marker that is used to detect early stages of IDA. RDW is often increased in cases of IDA as the bone marrow produces new smaller red cells into the circulation, thereby introducing heterogeneity in the size of the red cells population [20]. In this cohort, Ret-He surpassed RDW as the latter was not able to differentiate between ID and control group (RDW; *p* = 0.5 vs. Ret-He; *p* < 0.05). Tiwari et al. also used Ret-He with sTfR instead of RDW to detect latent iron deficiency (LID) in 501 blood donors with >92% sensitivity and >97% specificity [36]. A possible explanation behind the unchanged RDW in individuals with ID in our cohort is that while the iron store becomes depleted, the erythropoiesis continues the produce RBCs with normal size and hemoglobin content, as evidenced by the unchanged MCV and MCH when compared to the control group (MCV; *p* = 0.25, MCH; *p* = 0.47); hence, the bone marrow still produces a homogenous population of RBC, with only a small percentage (~1%) of the newly developed reticulocyte having a profound decrease in the hemoglobin content. This decrease, however, does not have a substantial effect on the MCV and MCH due to the minute percentage it represents.

The usefulness of Ret-He as a marker for detecting and diagnosing ID and IDA was further scrutinized by correlation studies and ROC analysis against the gold-standard non-invasive marker, serum ferritin as well as other iron markers. Ret-He showed a significant and positive correlation with the ferritin and TS and negative correlation with TIBC, similar to what has been reported by Toki et al. and Uçar et al. [34,35]. As a diagnostic marker, Ret-He promotes higher sensitivity and specificity with a cut-off of <21.55 and <28.25 pg for IDA and ID, respectively. These results are higher than what was reported previously. Uçar et al. used a Ret-He cut-off value of <25.4 pg to diagnose ID with a specificity < 90% and a sensitivity of 49.1% [35]. Toki et al. used a Ret-He cut-off value of 28.5 pg, similar to what was used in this study, to diagnose ID and had a specificity of >90%, while the sensitivity was 68%. At a higher cut-off (30.9 pg), the sensitivity increased to reach 92%, whereas the specificity to diagnose ID dropped back to 81% [34]. Tiwari et al. assessed the diagnostic usefulness of Ret-He to diagnose LID in blood donors with respect to sTfR and found that with a cut-off value < 28 pg, Ret-He had a higher sensitivity and specificity of 92% and 97%, respectively [36]. Brugnara et al. used a Ret He cutoff level of 27.2 pg to diagnose iron deficiency in hemodialysis patients with a sensitivity of >93%, and a specificity of >83% [17]. Together, the results from this cohort suggest that Re-He could be used with higher validity in detecting, diagnosing, and discriminating between ID and IDA cases.

Next, the efficacy of Ret-He in response to iron therapy was evaluated following the administration of 190 mg ferrous sulphate for a week. Brugnara et al. noted before that Re-He might be considered as an immediate marker to reflect the iron response therapy before the conventional ones [16]. In this study, Ret-He increased significantly in most of the participants with 7.5%, comparing to 1.3% increment in total Hb. This, in part, shows that Ret-He is very sensitive to iron incorporation and even a narrow window of time (7 days) is enough for the effect to be observed. It also demonstrates that the need to wait for several weeks/months to assess the treatment of choice might be overestimated and time consuming, especially in endemic areas of ID and IDA. This result is similar to what was reported by Uçar et al., where they measured the Ret-He after 5 days of the iron treatment (270 mg ferrous sulphate) and showed that Ret-He was increased and not Hb [35]. However, the exact increment for the combined group (ID and IDA) or each one was not specified. The favorable explanation behind the unchanged hemoglobin level even after the iron therapy is that it is detected through the cyanide-free, photometry method where the new and old RBCs are lysed. Hence, the iron incorporation occurred only in a very small subset of the newly developed RBCs and retics and therefore was not reflected in the total RBC population.

The limitations of this study are that it excluded pregnant women and participants suffering from malignancies and hemoglobinopathies. While this gives a better look into the usefulness of the marker in only the iron deficient population, it neglects, however, other conditions that might contribute to the development of anemia. This may explain the low number of participants in non-IDA group (*n* = 8) in this study. Future investigations should be directed toward examining the efficacy of Ret-He as a potential marker in the abovementioned diseases.

## 5. Conclusions

Together, the results from this study may push the medical communities, especially in endemic areas of IDA, toward utilizing Ret-He as potential marker for detecting and diagnosing different stages of ID with high validity. It also warrants Ret-He as a very sensitive and effective marker in reflecting the bone marrow response to iron therapy within short period of time. Future studies toward utilizing this marker in different erythropoietic disorders, such as hemoglobinopathies, anemia of chronic inflammation, anemia of infection, and sideroblastic anemia, are needed to establish reference ranges and suitable cut-offs for such conditions. Ret-He is a simple and integrated marker in most of the modern hematology analyzers and requires no additional tube.

## Figures and Tables

**Table 1:** Participants demographic data in all four groups.

Variable	Gender and Age	Control Group (*n* = 87)	ID Group (*n* = 55)	IDA Group (*n* = 32)	Non-IDA Group (*n* = 8)
		*n* (%)	*n* (%)	*n* (%)	*n* (%)
Gender	Male	75 (86.2)	8 (14.5)	0 (0)	1 (12.5)
	Female	12 (13.8)	47 (85.5)	32 (100)	7 (78.5)
Age	18–29	85 (97.7)	53 (96.4)	29 (90.6)	5 (62.5)
	30–49	2 (2.3)	2 (3.6)	3 (9.4)	1 (12.5)
	>50				2 (25)

Abbreviation: ID, iron deficiency; IDA, iron deficiency anemia; non-IDA, anemia without iron deficiency.

**Table 2:** Participants’ clinical findings.

	Parameters	Control (*n* = 87)	ID (*n* = 55)	IDA (*n* = 32)	Non-IDA (*n* = 8)	*p*-Value
Hematological Parameters	Hb (g/dL)	15.6 ± 1.8	13.3 ± 1.1	11.0 ± 0.8	10.7 ± 1.6	<0.001
	MCV (fL)	84.3 ± 4.9	85.5 ± 4.0	79.7 ± 7.6	82.9 ± 8.6	<0.0001
	MCH (pg)	28.8 ± 2.3	28.4 ± 1.7	25.0 ± 3.3	26.4 ± 3.9	<0.0001
	Ret-He (pg)	32.7 ± 2.7	31.7 ± 2.1	26.7 ± 4.5	29.0 ± 5.4	<0.0001
Iron Parameters	Ferritin (ng/mL)	71.1 ± 34.6	14.5 ± 7.8	8.7 ± 6.0	204.8 ± 288.0	<0.0001
	Serum Fe (umol/L)	16.9 ± 8.2	16.3 ± 15.3	8.0 ± 4.2	8.0 ± 6.8	<0.0001
	TIBC (umol/L)	60.1 ± 6.6	65.4 ± 6.8	66.4 ± 13.3	50.6 ± 11.6	<0.0001
	TS (%)	28.3 ± 13.7	25.8 ± 26.4	13.0 ± 8.9	0.9 ± 2.1	<0.0001

Results are shown as mean ± SD. For all the measurements, the *p*-value was obtained using Kruskal–Wallis test, and a *p*-value of <0.05 was considered statistically significant. Abbreviations: ID, iron deficiency; IDA, iron deficiency anemia; non-IDA, anemia without iron deficiency; Hb, hemoglobin; Ret-He, reticulocyte hemoglobin equivalent; MCV, mean cell volume; MCH, mean cell hemoglobin; serum Fe, serum iron; TIBC, total iron binding capacity; and TS, transferrin saturation.

## Data Availability

Data are contained within the article.
